# Enhancement of trichothecene mycotoxins of *Fusarium oxysporum* by ferulic acid aggravates oxidative damage in *Rehmannia glutinosa* Libosch

**DOI:** 10.1038/srep33962

**Published:** 2016-09-26

**Authors:** Zhen Fang Li, Chen Ling He, Ying Wang, Ming Jie Li, Ya Jing Dai, Tong Wang, Wenxiong Lin

**Affiliations:** 1 College of Crop Sciences, Fujian Agriculture and Forestry University, Jinshan, Fuzhou 350002, P.R. China; 2 College of Life Sciences, Fujian Agriculture and Forestry University, Jinshan, Fuzhou 350002, P.R. China

## Abstract

*Rehmannia glutinosa* is an important medicinal herb that cannot be replanted in the same field due to the effects of autotoxic substances. The effects of these substances on *R. glutinosa* in continuous cropping systems are unknown. In the present study, bioassays revealed that *R. glutinosa* exhibited severe growth restriction and higher disease indices in the FO+FA (*F.oxysporum* pretreated with ferulic acid) treatment. The increases in the contents of MDA and H_2_O_2_ were greater in the FA+FO treatment than in the FA or FO only treatments, respectively. Consistent with this result, the enzyme activities in the seedlings increased with treatment time. To identify the main factor underlying the increased pathogenicity of FO, macroconidia and trichothecene mycotoxins coproduced by FO were separated and used to treat *R. glutinosa* seedlings. The MDA and H_2_O_2_ contents were similar in the seedlings treated with deoxynivalenol and in the FA+FO treatment. Quantification of the relative expression of certain genes involved in Ca^2+^ signal transduction pathways suggested that trichothecene mycotoxins play an important role in the increased pathogenicity of FO. In conclusion, FA not only directly enhances oxidative damage in *R. glutinosa* but also increases wilting symptom outbreaks by promoting the secretion of trichothecene mycotoxins by FO.

*Rehmannia glutinosa* Libosch, an annual herb of the Orobanchaceae family, is one of the most common and important medicinal herbs^1^. The root tuber of this species is an important traditional Chinese medicine that is in high demand^2^. Fresh or dried tubers of *R. glutinosa* are mainly used to treat haematological conditions, insomnia and diabetes and for sedation^3^.

However, as an annual plant, *R. glutinosa* cannot be continuously cropped in the same field. In general, 8–10 years are required before *R. glutinosa* can be replanted in a field previously used for cultivation of this species^2^,^4^,^5^. Therefore, farmers must plant *R. glutinosa* in less desirable areas outside Jiao-zuo, inevitably resulting in a poor harvest in both yield and quality^3^,^6^.

An important obstacle to continuous cropping is autotoxicity, in which a plant inhibits the growth of plants of the same species by releasing autotoxic substances^7^,^8^. Autotoxic substances, such as ferulic acid (FA) produced by *R. glutinosa*, inhibit the growth of seedlings of the same species. These substances lead to oxidative stress due to the overproduction of reactive oxygen species (ROS), which cause membrane lipid peroxidation, protein denaturation and aggregation, DNA breaks and enzyme inactivation^9^. However, symptoms worse than those caused by autotoxicity have been noted in the field^4^,^10^.

Fungal diseases have recently attracted much attention as a factor limiting *R. glutinosa* cultivation^11^,^12^. *R. glutinosa* is prone to fungal diseases in monoculture systems, in which root rot and vascular wilt caused by FO is widespread and destructive^1^,^10^,^13^. Field observations have also revealed root rot and vascular wilt disease outbreaks, and 31 strains of *Fusarium* derived from soil under continuous *R. glutinosa* cropping were detected and identified using the plate method^10^.

Recent studies have increasingly focused on rhizospheric biological processes and plant-microbe interactions^14^,^15^,^16^. Several compounds present in root exudates or their degradation have been identified in autotoxicity processes and documented to shape rhizosphere microbiology by deterring or attracting certain microbial species^9^,^17^,^18^,^19^. Autotoxic substances play an important role in soil ecosystems and influence biochemical processes and plant fitness^19^,^20^,^21^.

Ca^2+^ ions play a vital role as second messengers in plant cells during different developmental processes and in various adaptation responses^22^,^23^. Accordingly, the currently held hypothesis is that Ca^2+^ signalling systems may be involved in oxidative damage^23^. The gene expression of certain Ca^2+^ sensors is enhanced in response to oxidative stress, facilitating their regulation and the ability of the cell to co-ordinate various signalling pathways.

In this paper, we focused on the interactions among autotoxic substances, microbes, and *R. glutinosa*. We first examined the direct effect of FA on *R. glutinosa* seedlings. Then, we indirectly assessed the effects based on the relationship between FA and FO. Finally, we quantified the relative expression of certain genes involved in Ca^2+^ signal transduction pathways.

## Results

### Both FA and FO inhibit the growth of R. glutinosa seedlings 7 days after inoculation

**Figure 1:**
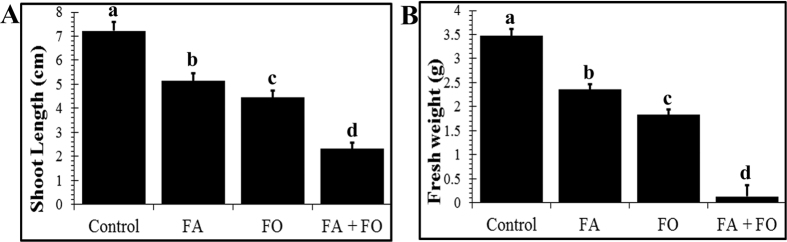
Both ferulic acid and *F. oxysporum* inhibit the growth of *R. glutinosa* seedlings. The shoot length (**A**) and fresh weight (**B**) of *R. glutinosa* seedlings at the 6-leaf stage were measured in response to treatment with nutrient solution (control), ferulic acid (FA), *F. oxysporum* (FO), or *F. oxysporum* pre-treated with ferulic acid (FA+FO). The data are presented as the mean of three independent replicates, with *n* = 6 plants per replicate. The error bars represent the standard error of the mean. Different letters indicate significant differences between groups according to one-way ANOVA followed by a post-hoc LSD test (*P* < 0.05).

Bioassays were performed to examine the ability of FA and FO to inhibit the growth of *R. glutinosa* seedlings. We first determined the concentration of FA (approximately 100 μmol·L^−1^) in a soil sample derived from a *R. glutinosa* continuous-cropping field. A significant decrease in shoot length was observed compared to the control upon treatment with FA (100 μmol·L^−1^) or FO (Fig. 1A). A similar decrease in fresh weight was observed (Fig. 1B), suggesting that both the FA and FO treatments led to growth retardation of *R. glutinosa* seedlings.

To investigate the effect of FA on FO, we incubated FO with FA prior to inoculation of the *R. glutinosa* seedlings. Interestingly, incubation with FA greatly enhanced the inhibitory effects of FO on *R. glutinosa* seedlings, with dramatic decreases in both shoot length and fresh weight (Fig. 1A,B).

### FA facilitates the pathogenicity of FO

**Figure 2:**
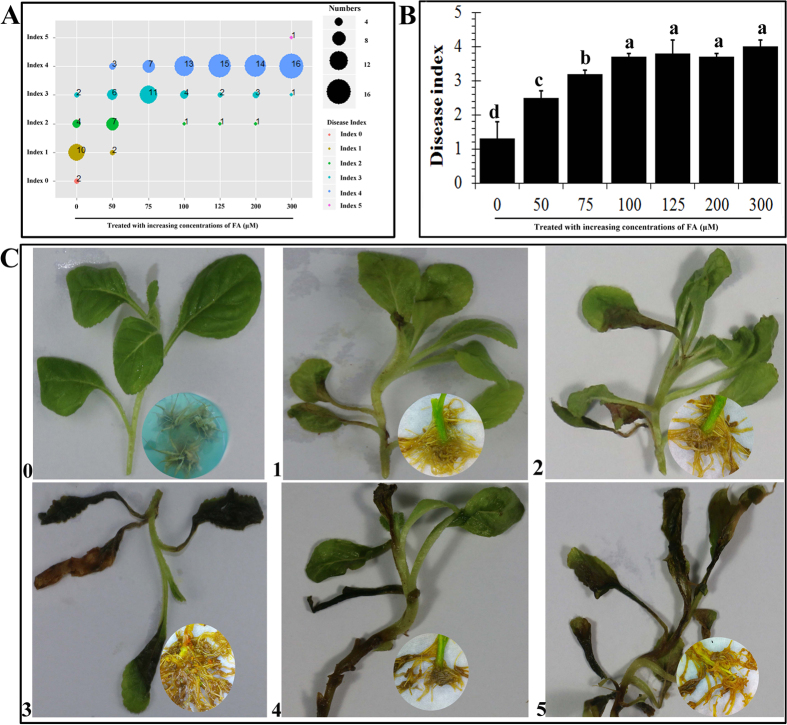
FA facilitates the pathogenicity of *F. oxysporum* on *R. glutinosa*. *F. oxysporum* cultured in nutrient solution at 1 × 10^4^ macroconidia per ml was treated withincreasing concentrations of FA for 2 days and was then used to inoculate 6-leaf-stage *R. glutinosa* plants. After 9 days, the plants with root rot and vascular bundle browning were counted and assessed a scale of 0–5 as follows: 0, healthy without any browning; 1, white shoot with scarce browning; 2, light shoot rot and browning; 3, mild shoot rot and browning; 4, severe shoot rot and browning; and 5, death of the whole plant. The statistical enrichment (**A**) for each treatment were demonstrated through the R Programming Language software. The mean disease index (**B**) for each treatment was tested. The data are presented as the mean of three independent replicates, with *n* = 6 plants per replicate. The error bars represent the standard error of the mean. Different letters indicate significant differences between groups according to one-way ANOVA followed by a post-hoc LSD test (*P* < 0.05). Photographs (**C**) of infected samples were the root rot symptoms and the vascular bundle browning symptoms which assessed at each scale.

Based on the results described above, we assumed that FA facilitates the pathogenicity of FO. To test this hypothesis, we measured the disease indices of *R. glutinosa* seedlings inoculated with FO that had been preincubated with FA at different concentrations (Fig. 2). The wilting symptoms of the inoculated plants were more serious when FO was pre-treated with FA, and all disease indices of the treatments were positive, demonstrating that the pathogenicity of FO was greatly enhanced by FA (Fig. 2). Compared to plants inoculated with FO (without FA treatment) as a control, mild vascular bundle browning was significantly enhanced by pretreatment of FO with FA. The browning index was 2.5 at 50 μmol·L^−1^ FA and reached 4.0 at 100 μmol·L^−1^ FA. Nearly all plants were dead at the end of the experiment. However, no further increases in the disease indexes of the *R. glutinosa* seedlings were observed at concentrations of FA of greater than 100 μmol·L^−1^ (100–300 μmol·L^−1^), consistent with the concentration of FA of approximately 100 μmol·L^−1^ in the soil sample derived from the *R. glutinosa* continuous cropping field^6^.

### Pretreatment of FO with FA induces greater oxidative damage in R. glutinosa seedlings

**Figure 3:**
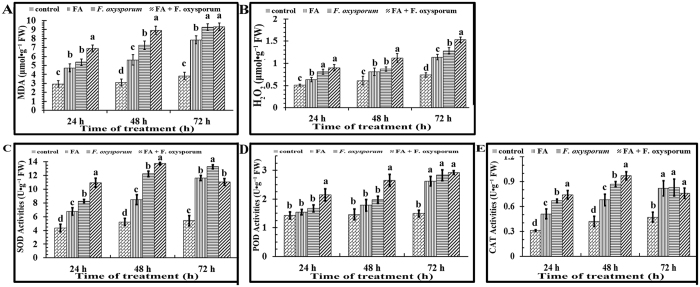
Oxidative damage and activities of protective enzymes increased in *R. glutinosa* plants treated with FA or *F. oxysporum.* Six-leaf-stage *R. glutinosa* seedlings were treated with FA, FO or FA+FO, and the contents of MDA (**A**), H_2_O_2_ (**B**), SOD (**C**), POD (**D**) and CAT (**E)** in the seedlings were measured at 24 h, 48 h and 72 h after treatment. FA: ferulic acid; FO: *F. oxysporum*; FA+FO: *F. oxysporum* pre-treated with ferulic acid (100 μmol·L−1). Each treatment contained 6 plants and was performed in triplicate. The error bars represent the standard error of the mean. Different letters indicate significant differences between groups according to one-way ANOVA followed by a post-hoc LSD test (*P* < 0.05). The statistical analyses were only conducted within a given time point; the statistical differences between time points were not considered.

Autotoxic compounds induce oxidative damage, which results in cellular membrane injury, solute and electrolyte leakage and membrane lipid peroxidation. FA treatment and FO inoculation (with or without FA pretreatment) of *R. glutinosa* resulted in increases in MDA and H_2_O_2_ content (Fig. 3A,B) and severe symptoms of autotoxicity. The seedlings treated with FA+FO exhibited the highest MDA and H_2_O_2_ content throughout the experiment, and the increases in MDA and H_2_O_2_ correlated well with the time since inoculation. For example, seedlings exposed to FA treatment for 1 day exhibited an 82.65% increase in MDA and a 45.24% increase in H_2_O_2_ (Fig. 3A,B).

To cope with oxidative damage, enzyme activities in the seedlings increased progressively when treated with FA and FO (with or without FA pretreatment) during the treatment period (days 1–3) compared to untreated seedlings. The SOD activity was higher in the FA‐or FO-treated seedlings compared to the control (Fig. 3C), and the SOD activity of the treated seedlings increased with time. The enzyme activities of the seedlings increased with treatment time, and the increases in enzyme activities became significant after 3 days of treatment. The SOD activity of the treated seedlings increased with FO treatment, and the trends of the activities of POD and CAT were similar to those of SOD, which increased with prolonged (days 1–3) exposure (Fig. 3D,E). Notably, the enzyme activities were higher in the seedlings treated with FA+FO than in the other treatments at 48 h after inoculation, which may indicate a corresponding increase in severe oxidative damage.

### FO mycelial growth and macroconidia numbers as well as production of trichothecene mycotoxins by FO are significantly increased after FA treatment

**Figure 4:**
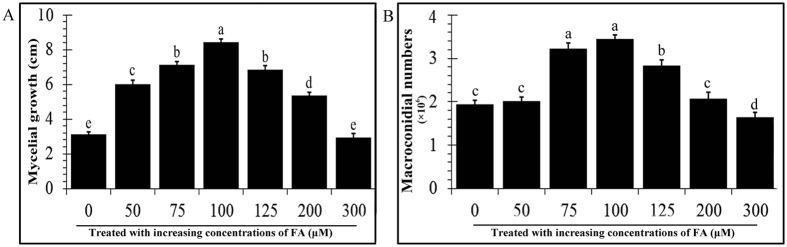
The mycelial growth and the number of macroconidia of *F. oxysporum* were significantly increased when treated with FA at a concentration of 100 μmol·L^−1^. (**A**) Agar plugs (0.7 cm diameter) of a 7-day-old colony of FO were placed in wells with different concentrations of ferulic acid. After a 72-h incubation at 26 °C in the dark, the colony diameter was estimated (cm). (**B**) FO (1 × 10^4^ macroconidia per ml) was cultured in liquid potato dextrose medium mixed with six different concentrations of FA solution. The sporulation of FO macroconidia in the six treatments was measured by a haemocytometer after incubation at 26 °C in the dark with shaking. Each treatment was performed in triplicate. The error bars represent the standard error of the mean. Different letters indicate significant differences between groups according to a one-way ANOVA followed by a post-hoc LSD test (*P* < 0.05).

To further determine the link between FA and FO, agar plugs (0.7 cm diameter) of a 7-day-old colony of FO were placed in the center of PDA agar plates containing different concentrations (0 μmol·L^−1^ control, 50 μmol·L^−1^, 75 μmol·L^−1^, 100 μmol·L^−1^, 125 μmol·L^−1^, 200 μmol·L^−1^, and 300 μmol·L^−1^) of FA. After 72 h of incubation at 26 °C in the dark, the colony diameter was estimated. The results showed that FA increased FO mycelial growth at concentrations from 50 μmol·L^−1^ to 100 μmol·L^−1^, but a drastic decrease in growth was observed after 3 days when the concentration of FA was greater than 100 μmol·L^−1^ (Fig. 4A). To display the effect of FA on the macroconidia production, 100 μl of spores of FO (10^4^ macroconidia per ml) were cultured in liquid potato dextrose medium mixed with different concentrations of FA solution described above. The numbers of sporulation of FO macroconidia in each treatment was measured after incubation using a haemocytometer. Compared to the control (untreated), macroconidia numbers were significantly higher from 50 μmol·L^−1^ to 100 μmol·L^−1^ FA, with the highest FO macroconidia number in the presence of 100 μmol·L^−1^ FA after 3 days (Fig. 4B).

**Figure 5:**
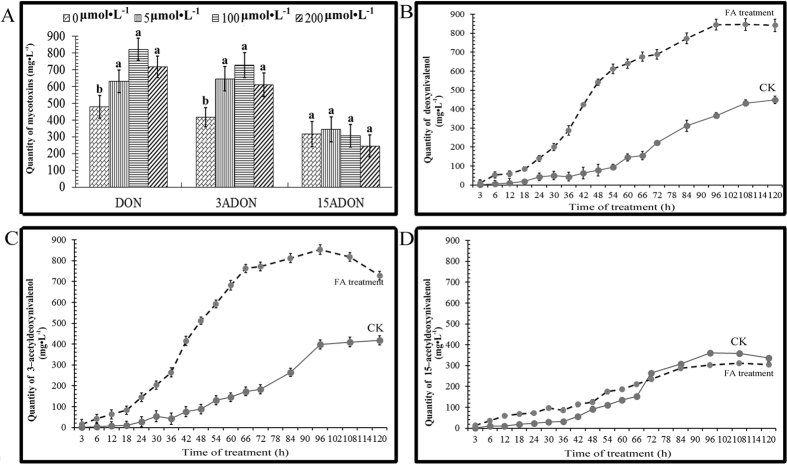
FA treatment increased the quantity of mycotoxins produced by *F. oxysporum*. (**A**) *F*. *oxysporum* (1 × 10^4^ macroconidia per ml) was treated with ferulic acid at concentrations of 0 μmol·L^−1^ (control), 50 μmol·L^−1^, 100 μmol·L^−1^, and 200 μmol·L^−1^ for 5 days, and the mycotoxins produced by *F*. *oxysporum* in each treatment were separated and quantified by HPLC. Production of the mycotoxins deoxynivalenol (**B**), 3-acetyldeoxynivalenol (**C**) and 15-acetyldeoxynivalenol (**D**) by *F. oxysporum* untreated (control) or treated with ferulic acid (100 μmol·L^−1^) was quantified by HPLC every 6 h after treatment. DON: deoxynivalenol; 3-ADON: 3-acetyldeoxynivalenol; 15-ADON: 15-acetyldeoxynivalenol. Each treatment was performed in triplicate. The error bars represent the standard error of the mean. Different letters indicate significant differences between groups according to a one-way ANOVA followed by a post-hoc LSD test (*P* < 0.05).

In addition, the production of trichothecene mycotoxin by FO was also evaluated with FA treatment (Fig. 5A). Deoxynivalenol (DON) and 3acetyldeoxynivalenol (3ADON) accumulation were higher after treatment with FA (at concentrations of 50 μmol·L^−1^, 100 μmol·L^−1^, and 200 μmol·L^−1^) compared to the control (untreated), with the highest production at 100 μmol·L^−1^ FA, these results consistent with the fact that concentrations of FA above 100 μmol·L^−1^ not only inhibit *R. glutinosa* growth (Fig. 1), but also FO mycelial growth and sporulation of FO macroconidia (Fig. 4). However, regardless of the concentration of exogenous FA, the quantity of 15-ADON (15-acetyldeoxynivalenol) did not increase.

We next focused on the dynamics of trichothecene mycotoxin production by FO. Trichothecene mycotoxin were collected and detected 120 h after treatment. DON and 3-ADON production were higher after treatment with FA compared to the control (untreated) and increased at an exponential rate in 48 h. However, DON and 3-ADON production was relatively slow in untreated FO (Fig. 5B,C). DON production by FO pretreated with FA was increased by more than 5.53-fold compared to the control. Similarly, 3-ADON was increased by more than 5.26-fold, up to 528 mg·L^−1^. Not surprisingly, production of 15-ADON was not obviously different from that of the control (Fig. 5D).

### Trichothecene mycotoxin plays a key role in the pathogenicity of FO

**Figure 6:**
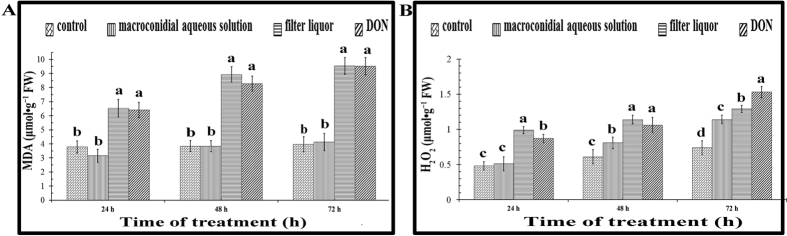
Trichothecene mycotoxin-containing filtered liquor as well as DON solution increased oxidative damage in *R. glutinosa* plants. Six-leaf-stage *R. glutinosa* plants were untreated, treated with filtered and resuspended macroconidia, filtered liquor (filtered culture supernatant) or DON solution, and the contents of MDA (**A**) and H_2_O_2_ (**B**) in each treatment were measured at 24 h, 48 h and 72 h after treatment. Each treatment was performed in triplicate. The error bars represent the standard error of the mean. Different letters indicate significant differences between groups according to a one-way ANOVA followed by a post-hoc LSD test (*P* < 0.05).

To determine if trichothecene mycotoxins are the main factor in the pathogenicity of FO macroconidia, we separated the trichothecene mycotoxins from the macroconidia. Spores produced by FO were diluted in a nutrient solution at 10^4^ macroconidia per ml after filtering the culture supernatant of FO; the filtrate without spores was also collected. The MDA and H_2_O_2_ contents of *R. glutinosa* seedlings treated with the macroconidia suspension or filtrate were then measured (Fig. 6). Treatment with the filtered liquor led to increases in the MDA and H_2_O_2_ contents, consistent with the trend in the contents of *R. glutinosa* seedlings treated with DON (500 mg·L^−1^). By contrast, there were no obvious increases in seedlings treated with the macroconidia suspension (Fig. 6), suggesting that the induced oxidative damage in *R. glutinosa* seedlings was due to increased trichothecene mycotoxins, not the increased mycelial growth or macroconidia production.

**Figure 7:**
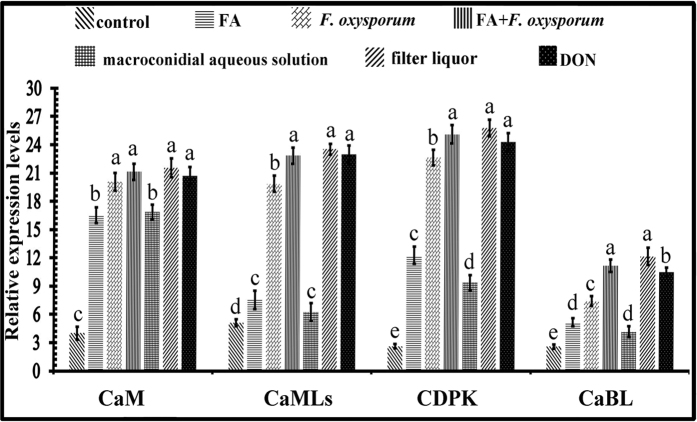
The relative expression of calcium pathway genes was enhanced by trichothecene mycotoxin treatment. The expression of the genes encoding calmodulin (CaM), calcium-dependent protein kinase (CDPK), calcineurin B-like protein (CaBL), and calmodulin-like protein (CaML) was measured in the untreated (control) group and groups treated with FA, *F*. *oxysporum, F*. *oxysporum* pretreated with FA, filtered and resuspended macroconidia, filtered liquor or DON solution. Gene expression was profiled by quantitative real-time RT-PCR using the comparative C_t_ method. Each treatment was performed in triplicate. The error bars represent the standard error of the mean. Different letters indicate significant differences between groups according to a one-way ANOVA followed by a post-hoc LSD test (*P* < 0.05). The statistical analyses were only conducted for a given target gene and that statistical differences between target genes were not considered.

We further studied the expression of Ca^2+^ sensors in plant signalling, including calmodulin (CaM), calcium-dependent protein kinase (CDPK), calcineurin B-like protein (CaBL), and calmodulin-like protein (CaML), in *R. glutinosa* seedlings treated with FA and FO. Quantitative real-time PCR revealed similar patterns of mRNA expression in all treatments with differential upregulation of these genes (Fig. 7). Compared to the controls, the transcript levels of CaM were significantly increased after FA or FO treatment by more than 8- to 10-fold. The transcript levels of CDPK exhibited a similar pattern after treatment, with an increase of more than 4- to 8-fold at 24 h after treatment. The expression levels of CaML and CaBL also increased significant and were more than 3-fold higher than in the control plants. Among these treatments, the FA+FO treatment, the filtered liquor treatment and the DON treatment exhibited similar expression levels of Ca^2+^ sensors. These results indicate that DON is the main factor underlying the pathogenicity of FO.

## Discussion

Continuous cropping obstacles are common phenomena for various crops, including greenhouse crops^21^,^24^, trees^25^,^26^, and, in particular, medicinal plants^27^,^28^. More than 70% of root-harvested medicinal plants, such as *Panax ginseng and* American Ginseng, can only be replanted once every 7–20 years due to these obstacles^26^,^29^.

The reasons for these obstacles remain unclear but include autotoxicity of root exudates and microbial community shifts. Greenhouse crops and leguminous crops tend to suffer from devastating fungal diseases or pests^21^,^24^, and medicinal plants and fruit trees are heavily impacted by autotoxicity issues^28^,^29^.

Both are considered key factors in the inability to continuously crop *R. glutinosa*. In the present study, *R. glutinosa* growth was affected not only by autotoxic substances but also by fungal diseases. Our field observations indicated that seedling growth was always inhibited by autotoxic substances at the early growth stage, whereas serious fungal diseases occurred in the late stage of *R. glutinosa* growth. Therefore, *R. glutinosa* provides a model for the study of the problem and its typical characteristics.

In the present study, FA had an inhibitory effect on the growth of *R. glutinosa* (Fig. 1). MDA and H_2_O_2_, indicators of oxidative damage, were enhanced by FA in the cells of *R. glutinosa* plants (Fig. 3). Similar increases in MDA and H_2_O_2_ have been reported in response to treatment with other phenolic compounds^30^,^31^. Moreover, FO inoculation, particularly its trichothecene mycotoxin, resulted in slight increases in MDA and H_2_O_2_ contents. We thus propose that both FA and FO trigger ROS generation and induce oxidative stress, disrupting cellular membrane structure and restraining seedling growth.

Furthermore, the activity and expression of most antioxidant enzymes are stimulated by ROS accumulation^10^,^32^. The accumulation of H_2_O_2_ in *R. glutinosa* in response to these treatments enhanced lipid peroxidation and caused severe oxidative stress, resulting in the disruption of metabolic activity in the cells. Antioxidant enzymes, such as CAT, SOD, and POD, remove accumulated H_2_O_2_. In the present study, increases in the activities of these antioxidant enzymes paralleled the accumulation of MDA and H_2_O_2_ in *R. glutinosa* plants after treatment. Increased POD activity in response to phenolic compounds has also been confirmed in cucumber roots^19^,^29^.

Autotoxin compounds shape rhizosphere microbiology by deterring or attracting certain microbial species^17^,^20^. In the present study, application of exogenous FA affected *F. oxysporum* mycelial growth, conidiophore production and trichothecene mycotoxin accumulation. Thus, FA produced by *R. glutinosa* not only significantly inhibits the growth of *R. glutinosa* plants but also significantly increases the incidence of root rot and vascular wilt. Indeed, previous investigations^13^ have reported that vascular wilt, a major soil-borne disease in many crops, is also promoted by exposure to autotoxins such as cinnamic acid, FA and 3,4-dihydroxybenzoic acid, suggesting an association of this disease with soil toxicity^8^,^30^,^33^.

In particular, our results demonstrate that treatment of *R. glutinosa* with FO leads to oxidative stress due to the overproduction of ROS. The effect observed is primarily attributable to the trichothecene mycotoxins, since that the possibility that that effect was due to increased mycelial growth was ruled out (Figs 6 and 7).

These results suggest that FA directly and indirectly exerts detrimental effects by triggering oxidative stress and disturbing seedling metabolism. In addition, FA promoted the production of DON by FO and thus led to oxidative damage in *R. glutinosa* plants via ROS generation, resulting in decreased plant growth and wilting symptoms. In conclusion, our results indicate that FA produced by fibrous roots plays an important role in soil-related disease outbreaks in monoculture systems.

Ca^2+^ ions play a vital role as second messengers in plant cells during various developmental processes and in response to environmental stimuli, acting as important sensors of Ca^2+^ flux in plants2^3^,^24^. The oxidative burst is another important component of pathogen defence. The present study, together with previously published data, reveals that the expression of certain Ca^2+^ sensor genes was altered in response to all the treatments. Hence, it is reasonable to suggest that Ca^2+^ signalling systems may be involved in oxidative damage processes.

Continuous cropping of *R. glutinosa* is a complicated issue, and further studies are needed to characterize additional signalling compounds involved in the *R. glutinosa-F. oxysporum* interaction.

## Materials and Methods

### Plant materials

The plant materials in this paper were micropropagated *R. glutinosa* (supplement Fig. S1), and the seedlings reached the 6-leaf stage and exhibited healthy roots without any physiological or morphological disorders^34^.

### Ferulic acid solution preparation

FA dissolved in ethanol was added to the nutrient solution at concentrations of 0 μmol·L^−1^, 50 μmol·L^−1^, 75 μmoL·l^−1^, 100 μmol·L^−1^, 125 μmol·L^−1^, 200 μmol·L^−1^, and 300 μmol·L^−1^. The final concentration of ethanol in each solution, including the control, was 0.1% (v/v), a concentration that has a negligible effect on *R. glutinosa* plants^8^ and has no effect on *F. oxysporum*^35^ (Supplement Table 1).

### *F. oxysporum* culture

A single strain of *F. oxysporum* (coded No. *CCS043*) was used for the fungal experiments and was isolated from soil under continuous cropping of *R. glutinosa* in Henan Province in China. The fungus was stored as a macroconidia suspension in 30% glycerol at −80 °C and was regularly transferred to growth plate cultures on potato dextrose agar medium (PDA) at 26 °C in the dark.

### Growth inhibition experiments of *R. glutinosa* seedlings

Using untreated healthy *R. glutinosa* micropropagated plants as a control, 3 micropropagated plants were treated with FA (100 μmol·L^−1^) for 2 days or *F. oxysporum* for 9 days. Each treatment was replicated 6 times in a completely randomized design.

In addition, the healthy *R. glutinosa* seedlings were inoculated with *F. oxysporum* that had been pretreated with FA (100 μmol·L^−1^). The untreated or treated seedlings were sampled at 24 h, 48 h, and 72 h; the tissue was frozen in liquid nitrogen and stored at −70 °C until analysis. At the end of the experiment (72 h after treatment), the seedling length and fresh weight were measured^32^.

### Pathogenicity test of *F. oxysporum* pretreated with ferulic acid

*F. oxysporum* cultured (PDA medium at 26 °C in the dark) in a nutrient solution at 1 × 10^4^ macroconidia per ml^36^ was treated with FA at concentrations of 0 μmol·L^−1^, 50 μmol·L^−1^, 75 μmoL·l^−1^, 100 μmol·L^−1^, 125 μmol·L^−1^, 200 μmol·L^−1^, and 300 μmol·L^−1^ for 2 days and was then used to inoculate *R. glutinosa* micropropagated plants. Each treatment contained 3 plants and was performed in 6 times. The experiment was terminated at 9 days after inoculation, when the FO-inoculated plants exhibited wilting symptoms with yellowing leaves. At the end of the experiment, each plant was harvested to assess root rot and vascular bundle browning on a scale of 0–5, as follows: 0, healthy without any browning; 1, white shoot with scarce browning; 2, light shoot rot and browning; 3, mild shoot rot and browning; 4, severe shoot rot and browning^8^; and 5, death of the whole plant.

### Measurement of MDA and H_2_O_2_

Lipid peroxidation was followed by measuring MDA accumulation using the method of Zhao *et al*. with some modifications^37^. Seedling samples (0.2 g) were homogenized in 0.1% trichloroacetic acid in phosphate buffer (5 ml, pH 7.8) and centrifuged at 12,000 rpm for 15 min. The supernatant (1 ml) was added to 0.5% thiobarbituric acid in 20% trichloroacetic acid (4 ml). The mixture was placed in a water bath at 100 °C for 10 min and then quickly cooled in an ice bath for 15 min. The samples were centrifuged at 12,000 rpm for 5 min, and the absorbance of the supernatant was measured at 450, 532, and 600 nm^37^.

H_2_O_2_ in the supernatant was measured according to Kang *et al*.^38^. Seedlings (0.5 g) were homogenized in 5 ml of pre-cooled acetone and centrifuged for 10 min at 1500 × *g*. Titanium chloride (0.1%, w/v) and concentrated ammonia (0.2 ml) were added to the supernatant (1 ml), and the mixture was allowed to react (10 min at 25 °C). The reaction mixture was then centrifuged at 1500 rpm for 10 min. The absorbance at 410 nm was measured, and the H_2_O_2_ concentration was calculated according to a standard curve.

### Extraction and assay of enzyme activities

Antioxidant enzymes (SOD, POD, and CAT) were extracted according to the method of Liu *et al*.^39^ and Sofo *et al*.^40^, with some modifications. Seedling samples (0.5 g) were homogenized in phosphate buffer (8 ml, 0.1 mol·L^−1^, pH 7.5) containing 2% (w/v) polyvinylpyrrolidone. The homogenate was centrifuged (12,000 rpm for 20 min), and the supernatant was used for enzyme analysis. All assays were performed at 2–4 °C.

Superoxide dismutase activity (SOD, E.C.1.15.1.1) was measured according to Liu *et al*.^39^ with minor modifications. The assay medium (3 ml) contained phosphate buffer (50,000 μmol·L^−1^, pH 7.8), EDTA–Na (100 μmol·L^−1^), *L*-methionine (12,000 μmol·L^−1^), riboflavin (2 μmol·L^−1^), and nitrotetrazolium blue chloride (75 μmol·L^−1^), Riboflavin was added last. The tubes were shaken and exposed to a photosynthetic photon flux of 50 μmol·m^−2^s^−1^ for 15 min. The reaction was initiated and terminated by turning the light on and off, respectively. The A_560_ was measured using a spectrophotometer, and the tubes containing the assay mixture, without the seedling extract (control), were illuminated to determine the maximum A_560_.

Peroxidase activity (POD, E.C.1.11.1.7) was measured according to Sofo *et al*.^40^. The reaction solution (3 ml) contained phosphate buffer (2.9 ml, 50,000 μmol·L^−1^, pH 7.0), guaiacol (50 μl, 10,000 μmol·L^−1^), H_2_O_2_ (10 μl, 40,000 μmol·L^−1^), and crude enzyme extract (40 μl). The increase in A_470_ due to the oxidation of guaiacol for 5 min was measured at 20 °C.

Catalase activity (CAT, E.C.1.11.1.6) was measured in a reaction mixture containing phosphate buffer (50,000 μmol·L^−1^, pH 7.0), H_2_O_2_ (30% w/v) and crude extract (10 μl). The breakdown of H_2_O_2_ was measured by following the decrease in absorbance at 240 nm for 2 min.

### Assessment of the effects of ferulic acid on *F. oxysporum* growth

The effects of FA on growth were evaluated on PDA medium in Petri dishes (diameter = 9 cm). After the medium was autoclaved, an ethanol solution of FA (filtered through a 0.22-μm filter membrane) was added, and the medium was thoroughly mixed and poured into Petri dishes^35^; the final concentrations of FA were 0 μmol·L^−1^, 50 μmol·L^−1^, 75 μmol·L^−1^, 100 μmol·L^−1^, 125 μmol·L^−1^, 200 μmol·L^−1^, and 300 μmol·L^−1^ 19.

Individual wells (0.7 cm diameter) were formed in the plates by cutting a square in the centre of the plates. Then, an agar plug (0.7 cm diameter) of a 7-day-old colony of *F*. *oxysporum* was placed in the well. After 72 h of incubation at 26 °C in the dark, the colony diameter was estimated (cm). The experiments for each condition were performed in triplicate and were independently replicated 6 times^9^,^41^.

### Assessment of the effects of ferulic acid on macroconidia production

As described by Ling *et al*.^36^, the sporulation of *F*. o*xysporum* macroconidia was induced in liquid potato dextrose culture at 26 °C in the dark. After 10 days, the spores were carefully filtered through two layers of sterile lens paper to eliminate mycelial fragments; Counts were determined in liquid potato dextrose cultures using a haemocytometer. Then, 100 μl of spores of *F. oxysporum* (diluted into 1 × 10^4^ macroconidia per ml) was cultured in liquid potato dextrose medium mixed with different concentrations of FA solution^42^,^43^. The final concentrations of FA are described above. The germination assay was performed at 26 °C in the dark with shaking at 140 rpm for 72 h^43^; There were three replicates for each treatment. The sporulation of *F*. *oxysporum* macroconidia in the 6 treatments (5 concentrations of FA and one control at 0 μmol·L^−1^ FA) was measured after incubation using a haemocytometer.

### Assessment of the effects of ferulic acid on trichothecene mycotoxins

For the hyphal growth and spore-bearing experiments, 4 treatments were designated as follows: control (0 μmol·L^−1^ FA); 50 μmol·l^−1^ FA; 100 μmol·L^−1^ FA; and 200 μmol·L^−1^ FA. For the mycotoxin analyses, *F*. *oxysporum* macroconidia were selected. Macroconidia suspensions (10^4^ macroconidia per ml) were generated in the liquid potato dextrose medium and were mixed with FA solution in Erlenmeyer flasks^44^. The cultures were incubated at 26 °C in the dark with shaking at 200 rpm. The toxin levels in the treatment are expressed in micrograms of toxin per litre of liquid medium after 5 days of control culture. Each treatment was replicated 6 times in a completely randomized design.

### Quantification of trichothecene mycotoxins produced by *F. oxysporum*

The macroconidia cultures were stopped at the point time and filtered through two layers of sterile lens paper. The DON, 3-ADON, and 15-ADON mycotoxins were extracted from the filtrates using 3-fold volumes of ethyl acetate according to the protocol described by Ponts *et al*.^18^. The samples were shaken vigorously, sonicated on ice, and allowed to stand for 30 min for phase separation. Thereafter, the organic phase was evaporated to dryness at 70 °C under a nitrogen stream. The dried samples were re-dissolved in 200 μl of methanol/water (50%, vol/vol) before analysis by HPLC^44^.

To determine the levels of trichothecene mycotoxins produced by *F*. *oxysporum*, DON, 3-ADON, and 15-ADON mycotoxins purchased from Sigma (HPLC grade) were used as standards. Quantification was performed with external calibration using standard solutions of DON and 15-ADON prepared from commercial pure powders (Sigma-Aldrich. Saint Louis, Missouri 63103 USA).

According to the protocol described by Boutigny *et al*.^4^ and Ponts *et al*.^18^,^44^, the mycotoxins extracted from the different treatments were analysed using a Waters 2695 HPLC system with a 250 × 4.6 mm Luna 5 μm C18 100 Å column (Phenomenex, Torrance, CA). A photodiode array (PDA) detector was used with an isocratic solvent system [methanol: water-methanol containing 5% (v/v) (90:10) ratio]. The PDA detector measured the UV spectrum (190–500 nm). The samples were dissolved in acetonitrile, and 10 μl was loaded onto the column using an automatic injector. The mycotoxins were eluted with solvent or the mobile phase at a rate of 0.75 ml·min^−1^ for 25 min. Standard curves for the respective mycotoxins were generated based on 5 different concentrations of pure toxins, and the absorbances obtained from the HPLC analyses. The mycotoxins produced by the same population in each treatment were sampled over time and replicated 3 times in a completely randomized design.

### Autotoxin and trichothecene mycotoxin treatments

When the *R. glutinosa* seedlings reached the 6-leaf stage with a healthy root, batches of uniform seedlings were transferred to the seedling medium; 3 micropropagated plants were transplanted into glass growth vessels, and each treatment was performed six times. The seedlings were allowed to acclimate to the hydroponic conditions for 7 days.

The medium of *F. oxysporum* pretreated with 100 μmol·L^−1^ FA was extracted to evaluate the effects of FA on trichothecene mycotoxins. Based on previous experiments investigating the effects of FA on trichothecene mycotoxins^29^, we designated 4 treatments: control (0 μmol·L^−1^ FA solution); FA (100 μmol·L^−1^ FA solution); FO (extract of the *F*. *oxysporum* medium without pretreatment with FA); and FO+FA (extract of the *F*. *oxysporum* medium pretreated with 100 μmol·L^−1^ FA). Five millilitres of each of the solutions and the extracts, which were filtered through a 0.45-mm filter, was added to the robust seedling medium for the experiment, and each treatment was replicated 6 times in a completely randomized design. The seedlings were added with an equivalent volume of distilled water and ethanol as the control. All glass growth vessels were maintained in a tissue culture room at 26 °C with fluorescent lights for 11 h (8:00–20:00), and the fluorescent light intensity was 4.17 ± 0.18 × 10^3^ lux^10^.

### Quantitative real-time RT-PCR (qRT-PCR) analysis of calcium pathway genes

To quantify calcium pathway gene expression, total RNA was isolated from *R. glutinosa* seedlings in the FA (100 μmol·L^−1^ FA), FO (*F. oxysporum*), and FO + FA (*F. oxysporum* pretreated with 100 μmol·L^−1^ FA) treatments.

qRT-PCR analysis was performed using a One-step Quanti-Tect SYBR Green RT-PCR Kit (Qiagen, Shanghai, China). The 18 S rRNA gene was used as the internal control. The primer pairs are listed in Table 1^45–48^.

The PCR reactions were performed according to the manufacturer’s instructions. The RT-PCR conditions were 1 cycle of 95 °C for 5 min; 40 cycles of 95 °C for 10 sec, 59 °C for 30 sec, and 72 °C for 30 sec; and 1 cycle of 72 °C for 7 min. The data were analysed using the comparative C_t_ method.

### Statistical Analysis

All data were subjected to analysis of variance using the Statistical Analysis System Program (SPSS 9.0). Each value was expressed as the mean of 3 replicates ± the standard error (SE). Statistical significance was analysed using Student’s *t*-test and one-way analysis of variance (ANOVA). The precision of the method was confirmed by least-significant difference (LSD, %). The values were considered significant when the *P* value was < 0.05.

## Additional Information

**How to cite this article**: Li, Z. F. *et al*. Enhancement of trichothecene mycotoxins of *Fusarium oxysporum* by ferulic acid aggravates oxidative damage in *Rehmannia glutinosa* Libosch. *Sci. Rep.* **6**, 33962; doi: 10.1038/srep33962 (2016).

## Supplementary Material

Supplementary Table S1

Supplementary Figure 1

## Figures and Tables

**Table 1:** Gene and qRT-PCR primers used in this study.

Genes	Primers (5′ to 3′)
Calmodulin (CaM)	F: GAGAAGAGGCACCCTTTGTTT
	R: TGGTATATGGGTTGCTGTTGG
Calmodulin–like protein (CaML)	F: TAGATCCTCACCACTGCAACC
	R: ACCTGAAGATGGACCCAAAAC
Calcium–dependent protein kinase (CDPK)	F:AAAGAAAAGCCTCGCAAACTC
	R: TAAGCGATAAACGCAAATGCT
Calcineurin B–like protein (CaBL)	F: TTAGAGGGGATCAAGCATTTATGTG
	R: ATCAGCCCGTCATCAATCACAGC
Internal control	F: GTTCTTAGTTGGTGGAGCGATT
	R: CAGACCTGTTATTGCCTCAAAC
