# Supplementary material for: Enhancement of trichothecene mycotoxins of Fusarium oxysporum by ferulic acid aggravates oxidative damage in Rehmannia glutinosa Libosch
Source: Sci Rep. 2016 Sep 26;6:33962. doi: 10.1038/srep33962 (PMC5036203; doi:10.1038/srep33962)
Supplement: Supplementary Table S1 [file srep33962-s1.doc]

**Enhancement of trichothecene mycotoxins of *Fusarium oxysporum* by ferulic acid aggravates oxidative damage in *Rehmannia glutinosa* Libosch**

Zhen Fang Li^1^*, Chen Lin He^1^*, Ying Wang^2^, Ming Jie Li^1^, Ya Jing Dai^1^, Tong Wang^1^, Wenxiong Lin^2^**

^1^College of Crop Sciences, Fujian Agriculture and Forestry University, Jinshan, Fuzhou 350002, P.R. China

^2^ College of Life Sciences, Fujian Agriculture and Forestry University, Jinshan, Fuzhou 350002, P.R. China

**Supplement *Table 1*. Ethanol present in the FA preparations has no effect on *F. oxysporum.***

^*^ Means these authors contributed equally. ^**^Correspondence author: Wenxiong Lin

Correspondence email: sxlizhenfang@126.com

College of Life Sciences, Fujian Agriculture and Forestry University, Jinshan, Fuzhou 350002, P.R. China

Phone: 86‐591‐83722796 Fax: 86‐591‐83789440

**Supplement *Table 1*. Ethanol present in the FA preparations has no effect on *F. oxysporum***

| **Concentrations of FA**  **（μmol•L^-1^）** |  | **Mycelial growth**  **（cm）** |  | **Macroconidia numbers**  **(×10^6^)** |  | **The quantity of mycotoxins (mg•L^-1^)** | | |
| --- | --- | --- | --- | --- | --- | --- | --- | --- |
|  |  |  |  |  |  | **DON** | **3-ADON** | **15-ADON** |
| **Control ^1x^** |  | 3.11±0.35 |  | 1.93±0.11 |  | 448±19.2 | 418±21.4 | 337±20.8 |
| **Control ^2y^** |  | 3.20±0.34 |  | 1.79±0.15 |  | 451±21.1 | 420±20.6 | 341±19.9 |
| **50** |  | 6.01±0.42 |  | 2.01±0.29 |  | 632±20.1 | 646±25.2 | 345±22.1 |
| **100** |  | 8.44±0.38 |  | 3.44±0.30 |  | 841±33.2 | 728±21.5 | 306±18.3 |
| **200** |  | 5.37±0.28 |  | 2.07±0.25 |  | 718±19.6 | 610±20.3 | 245±31.2 |

**X Control ^1^ no contained ethanol and was used as control for FA ( 0 μmol•L^-1^）.**

**Y Control ^1^ contained** 0.1% (v/v) **ethanol and was used as control for FA ( 0 μmol•L^-1^）.**
