# Supplementary material for: Enhancement of trichothecene mycotoxins of Fusarium oxysporum by ferulic acid aggravates oxidative damage in Rehmannia glutinosa Libosch
Source: Sci Rep. 2016 Sep 26;6:33962. doi: 10.1038/srep33962 (PMC5036203; doi:10.1038/srep33962)
Supplement: Supplementary Figure 1 [file srep33962-s2.doc]

**Enhancement of trichothecene mycotoxins of *Fusarium oxysporum* by ferulic acid aggravates oxidative damage in *Rehmannia glutinosa* Libosch**

Zhen Fang Li^1^*, Chen Lin He^1^*, Ying Wang^2^, Ming Jie Li^1^, Ya Jing Dai^1^, Tong Wang^1^, Wenxiong Lin^2^**

^1^College of Crop Sciences, Fujian Agriculture and Forestry University, Jinshan, Fuzhou 350002, P.R. China

^2^ College of Life Sciences, Fujian Agriculture and Forestry University, Jinshan, Fuzhou 350002, P.R. China

Supplement *Fig S1* title: The Materials of the micropropagated *R. glutinosa* seedlings.

^*^ Means these authors contributed equally. ^**^Correspondence author: Wenxiong Lin

Correspondence email: sxlizhenfang@126.com

College of Life Sciences, Fujian Agriculture and Forestry University, Jinshan, Fuzhou 350002, P.R. China

Phone: 86‐591‐83722796 Fax: 86‐591‐83789440

**Supplement *Figure 1*. The Materials of the micropropagated *R. glutinosa* seedlings.**

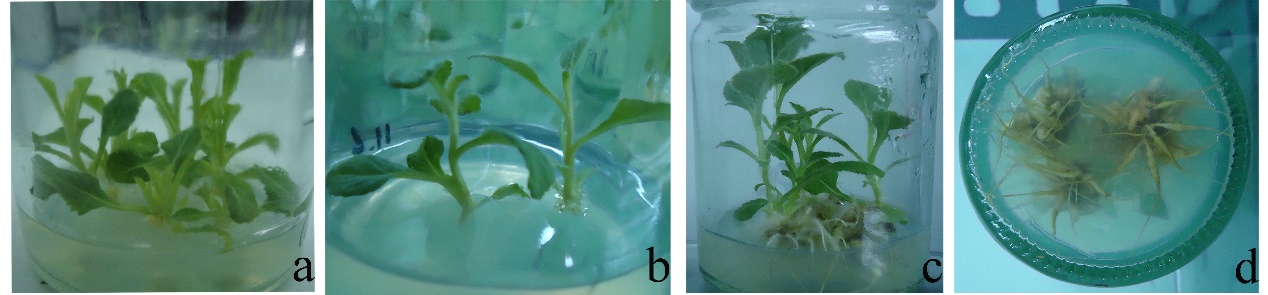


**a** Shoot tips of *R. glutinosa* were surface sterilized in a 0.5% sodium hypochlorite solution for 10 min and then cultured in inducement medium for 2 weeks.

**b** The shoot tips were transferred to propagation medium for 4 weeks; after multiple buds formed

**c** The samples were transferred to rooting medium

**d** After 3 weeks of culture, the micropropagated *R. glutinosa* seedlings reached the 6-leaf-stage and exhibited healthy roots.
